# Sociological factors influencing the performance of organic activities in Iran

**DOI:** 10.1186/s40504-020-00098-z

**Published:** 2020-05-11

**Authors:** Mahsa Fatemi, Kurosh Rezaei-Moghaddam

**Affiliations:** Department of Agricultural Extension and Education, School of Agriculture, Shiraz University, Shiraz, Iran

**Keywords:** Organic agriculture, Environmental sociology, Attitude, Pro-environmental behaviors

## Abstract

The conventional production model based on extensive use of chemical inputs such as pesticides is increasingly challenged. Organic agriculture is considered as one of the most important alternative agricultural systems to produce healthy food without any chemicals. Current models are not suitable for prediction of environmental behaviors. The current study aims to analyze the diffusion of organic agriculture to produce healthy food with the environmental sociology approach among farmers. The study was conducted using the survey research and multi-stage random sampling in Fars province, in the south of Iran. The samples included 215 farmers. The reliability of the questionnaire was confirmed by conducting a pilot study out of the main sample. The results showed that the farmers have strong attitude to the environment and are for the most part, highly intentioned to perform organic activities. Based on the results, the farmers’ intention toward adoption of organic agriculture, environmental identity, and responsibility of pro-environmental behavior, and their moral norms play an important role to accept organic agricultural activities. There are also some effective factors in implementation of organic agriculture including farmers` increasing awareness about the principles of organic farming, controllability of environmental behaviors as well as their accessibility to the resources and facilities for organic farming. The study emphasized that the attitude and enhancing the knowledge do not lead to pro-environmental behaviors and organic activities directly. Using the models and environmental sociology theories is more efficient to explain pro-environmental behaviors. To this aim, some suggestions were presented to increase the adoption of organic activities and persuade the Iranian farmers to select this kind of cultivation.

## Introduction

Since the green revolution in the 1950s, the traditional agriculture system has moved toward the green agriculture or the conventional agriculture system. Green agriculture aimed to produce more agricultural products in order to supply food for the world’s people. Alongside increasing the agricultural products and modifying the food shortage, a large number of problems have been raised in agricultural ecosystems of developed and developing countries. Therefore, the current production model based on using high chemical inputs such as pesticides aiming to increase agricultural productions is challenging since a large number of studies demonstrated destructive impacts on the environment and negative impacts on producers and consumers’ health (Gracia and de Magistris 2013). Some problems include the contamination of water sources, affecting the soil’s health, and decreasing absorbable amount of some micronutrients such as zinc, iron and copper leading to the loss of biological balance of ecosystems, consequently, pests’ resistance to pesticides and a rise in new pests, as well as reducing the quality of agricultural products (Malek-Saeidi et al. 2012). Observing the undesirable effects of the conventional agriculture on the world results in emphasizing the immediate need for developing agricultural techniques which are sustainable environmentally and socio-economically (Rezaei-Moghaddam et al. 2005). Many concerns are observed about the effects of some agricultural activities on the environment and society in the last years. Agricultural policies in many countries have moved toward a staunch friendship with nature. To this aim, the organic agriculture is considered as one of the most important alternative agricultural systems to produce healthy food without any chemicals (Klockner 2013).

The organic agriculture looks for the ecosystems which can control pests and weeds alongside sustainable utilization of land. The organic agriculture techniques are based merely on respecting nature, protecting the environment, and utilizing it sustainably (Nemat Pour and Rezaei-Moghaddam 2014). Organic agriculture includes comprehensive production management systems. The systems employ the management activities which improve the health of agroecosystem such as biodiversity, biological cycles, and biological activities of soil by emphasizing the use of existing inputs in farm and biological and mechanical methods as far as possible instead of synthetic chemicals (Rai 2005).

The pro-environmental behavior is regarded as the least harmful behavior or as useful as possible for the environment (Chen 2015; Pradhananga et al. 2017). The researchers investigated the pro-environmental behaviors in various and specific levels. Selecting the behaviors which significantly affect the environment is the first step for developing behavioral changes (Steg and Vlek 2009). Distinction between various levels of pro-environmental behavior plays a critical role in determining and prioritizing the behaviors which have the most effect on behavior quality. The pro-environmental behaviors are always presented as “selective logical conditions”. It means that people evaluate the pros and cons of an action and choose an option which has the most personal benefit (Harland et al. 2007). The rational selection approach is based on this assumption that “people eventually act logically;” since “they use available information rationally”, “are not controlled by unconscious motives or instinctive desires” and do not act without thinking (Ajzen and Fishbein 1980; Ajzen and Madden 1985). Based on the pro-environmental behavior model, environmental knowledge leads to environmental consciousness and attitude, which results in a pro-environmental behavior. This rationalist model assumes that educating the people on environmental issues leads to the outbreak of pro-environmental behavior (Rigby and Cáceres 2001). The primary models of rational selection were extremely simple, in a way that environmental knowledge and consciousness, as well as environmental concerns affect pro-environmental behavior via linear models. These models assume that education could strengthen pro-environmental behavior. However, the literature review demonstrates that the relationship between knowledge and behavior is weak. Despite confirming the relation between attitude and behavior, a direct relation between knowledge and attitude was not found (Shanks 2006). Other studies concluded that intentions based on high knowledge predict the behavior better than those based on low knowledge (Fabrigar et al. 2006). In addition, this model is highly simplistic which does not consider the other variables (Kollmuss and Agyeman 2002). However, a large number of non-governmental organizations develop their strategies based on this simple model and claim that more environmental knowledge leads to the outbreak of pro-environmental behaviors. However, relying on knowledge to create change is rather strange since changing the behavior is quite complicated.

By understanding the relationship between attitude and behavior, the psychologists seek to present the models which demonstrate components of attitude and behavior precisely (Banytė et al. 2015). Stryker and Burke (2000), by presenting a simple model, claim that knowledge, attitude, and behavior are related to each other. It means that knowledge impacts attitude and leads to the outbreak of a particular behavior. The Planned Behavior theory is probably the most effective rational selection model in social psychology. This model presents a logic framework, which investigates the effect of the variables such as attitude, subjective norms and Perceived Behavioral Control (PBC) on pro-environmental behavior (Lane and Potter 2007). The Theory of Planned Behavior (TPB) is the evolved theory of reasoned action. The reasoned action theory is based on the assumption that behaviors are under people’s “voluntary control”. However, there are behaviors which people have limited voluntary control on. The Planned Behavior theory compensates the limitations of reasoned action theory by presenting the criteria of behavior control. Another model is the Responsible Environmental Behavior (REB), which is based on Ajzen and Fishbein’s Planned Behavior theory. According to this model, intention to perform a behavior is a determining factor for outbreak or non-outbreak of a particular behavior. This model assumes that attitude, locus of control, and responsibility are the factors which lead people to do a behavior. In addition, people’s knowledge on environmental issues and functional strategies, as well as people’s skill of pro-environmental behavior play a critical role in developing a pro-environmental behavior (Kollmuss and Agyeman 2002).

Schwartz proposed Norm Activation model, in which moral norms and self-expectation to outbreak of socialist behavior direct people’s humanitarian behaviors. The Norm Activation model assumes that people act in a manner which is congruent with their values (Klockner 2013). Based on this theory, people are more likely to develop the sense of personal commitment in themselves, when they are aware of the consequences of their behavior. Activating the personal norm needs people’s responsibility for contributing (local or positional responsibility). In this regard, distinction between two different norms (social and personal) is significant. Expectations and limits of social norms depend on the social environment, while those related to personal norms depend on the people. Social norms could influence personal norms and behaviors if people regard them as a basis for self-evaluation (Pradhananga et al. 2017). Based on this theory, the personal norms are the key factors, which affect pro-environmental behavior directly (Phipps et al. 2013).

Value-Belief-Norm (VBN) theory presents a casual chain of determinants in pro-environmental behavior (Huffman et al. 2014; Lane and Potter 2007; Raymond et al. 2011; Phipps et al. 2013) which moves from “consistent elements of personality and belief” to “more focus on unpleasant consequences of values goals and people’s responsibility” for decreasing the risks. This theory assumes that individual’s moral norms are activated when people are aware of unpleasant consequences of particular environmental conditions, which may threaten the people’s desired values. Therefore, people feel responsibility to decrease the unpleasant consequences (Stern et al. 1999; Stern 2000). Value-Belief-Norm theory involves Norm Activation model, New Ecology Paradigm (NEP) and Personal Values (Phipps et al. 2013; Lopez-Mosquera and Sanchez 2012; Klockner 2013; Raymond et al. 2011; Lo et al. 2012).

Analysis of different theories and models indicates that attitudes and beliefs may not lead in a straightforward way to environmental behaviors. Social factors influence individual attitude and intention and thereby impact behavior toward environment. Prediction and improvement of behaviors related to environment should be and appear to be, evolving as a universal priority. Therefore, the study of environmental attitudes and behaviors is a vitally important task for the scientific community. Contemporary advances in environmental consciousness provide a multitude of opportunities for research and new knowledge generation in topics of interest to environmental sociologists and environmental scientists. Thus, a more robust and systematic sociological study of environmental issues seems to be universally meritorious and very timely. Conventional models fail to incorporate the dynamics of interactions between and impact of individual and communal human behavior and the environment. Environmental sociology investigates complicated and diverse symbolic and non-symbolic interactions and reciprocal influences between society and environment, which includes not only social and cultural aspects, but also physical and biological ones (Rezaei-Moghaddam et al. 2005; Rezaei-Moghaddam and Karami 2008). Specifically, environmental sociology concentrates on the reciprocally influential relationships between the environment-social and physical-and human behavior (Hughes 1995). In light of advancements in the field of environmental sociology, Rezaei-Moghaddam and Fatemi (2013) suggest a new modeling approach and provide a more robust and comprehensive tool with better resolution. Based on this model, awareness of environmental consequences, manifestation of pro-environmental behavior responsibility, environmentally-based social affects, environmental knowledge, social norms, environmental identity and controllability of behaviors are the main determinants of the model.

**Fig. 1:**
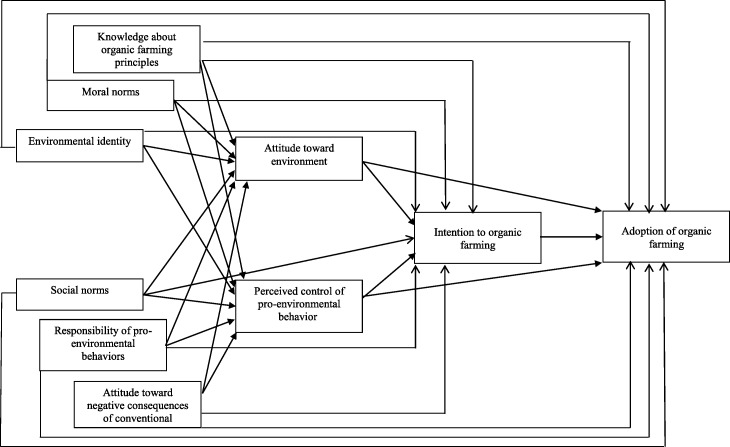
Conceptual framework of adoption behavior analysis of farmers about organic agricultural activities

The overview of research model has been presented in Fig. 1. In this model it is assumed that the independent variables are the main and direct factors in adoption of the organic agricultural activities. These variables include knowledge of organic agriculture principles, responsibility of pro-environmental behavior, environmental identity, attitude to negative consequences of the conventional agriculture, social norm, moral norm, attitude to the environment, perceived control of pro-environmental behavior and intention to adopt organic agricultural activities. In addition, the presented model in the study assumes that these variables affect the adoption of organic agricultural activities indirectly, which by paying attention to indirect effects of these variables enriches the analysis of the relationship between these variables and organic agricultural behavior.

Despite many benefits of organic agriculture, official statistics demonstrate that only 5% of farmers in Iran cultivate organic products and this is highly disappointing (Masoud 2017). Based on global statistics, the area under cultivation of organic products is 50,900,000 ha totally. The amount is 22,800,000 in Oceana, 12,700,000 in Europe, 6,700,000 in Latin America, 4,000,000 in Asia, 3,000,000 in North America and 1,700,000 in Africa. The area under cultivation of organic products has increased 14.7% during the last years. Australia, Argentina, United States, Spain, China, Italy, Uruguay, India, and Germany are pioneers in organic agriculture. The area under cultivation of organic products is 22,700,000, 3,000,000, and 2,000,000 in Australia, Argentina, and the United States, respectively (IFOAM 2017). However, despite potential capacity and possibility of attendance in global markets, the Iran share of these areas is highly low. The last organic statistic in this country in 2014 is 34,450 ha (Damghani 2016). Given this issue, Iran, by relying on its potential capacities, should move toward sustainable agriculture through organic activities seriously, and this movement is not possible without contributing all involved factors.

A large number of Iranian agricultural specialists criticize the lack of respect for pro-environmental activities. They focused on creating a new agricultural approach for achieving sustainable development in order to stop unnecessary consumption of chemicals, which harm the environment, farming areas and human health considerably, as well as using more organic productions since providing the healthy food and minimizing the environmental contamination arising from the use of agricultural pesticides are the general policies and strategies in Iran (Rezaei-Moghaddam and Fatemi 2013; Rezaei-Moghaddam et al. 2005). Current study aimed to analyze the development of organic agriculture for producing healthy food with the environmental sociology approach among Iranian farmers.

## Research method

**Fig. 2:**
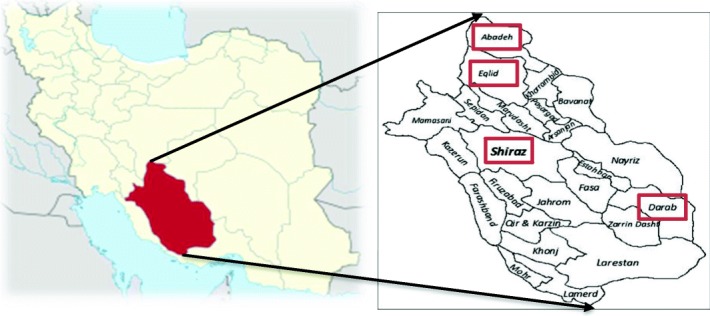
Geographical situation of studied area

The current study was conducted by survey research in Fars province in the south of Iran (Fig. 2). The statistical population included the farmers who adopted the organic activities. Estimating the research sample using multi-stage random sampling, in the first step, 21 villages from the four counties of *Shiraz, Abadeh, Eqlid and Darab* of Fars province were selected, and then 10–15 rural households were chosen from each village, randomly. Finally, 215 farmers were interviewed in person due to the formula 1 (Fowler 2009).

$$ \mathrm{n}=\frac{\mathrm{N}\ {\partial}^2}{\left(\mathrm{N}-1\right)\ \mathrm{D}+{\partial}^2} $$

where:

*N* = Total number of the villages in research area.

*n* = Sample size.

∂^2^ = Variance of the sample (using the results of the pilot study).

*B* = Estimated error which is assumed 1 in this study.

**Formula 1.** Formula of the estimation of sample size.

The data were collected by the questionnaire. The validity and reliability of the questionnaire were confirmed by professors and experts in this field as well as a pilot study out of the main sample (Cronbach’s Alpha = 0.65 and 0.93) (Table 1). Table 2 indicates the definition of the main variables.

**Table 1:** Cronbach’s alpha coefficients of research variables

Cronbach’s alpha	Variables
Environmental identity	0.82
Intention for organic agriculture	0.77
Knowledge of organic agriculture principles	0.84
Adoption of organic agricultural activities	0.93
Attitude toward the environment	0.75
Perceived control of pro-environmental behaviour	0.65
Social norms	0.81
Responsibility of environmental behaviour	0.78
Moral norms	0.90
Attitude to negative consequences of the conventional agriculture	0.86

**Table 2:** Definition of the research variables

Type of Variable	Variables	Definitions
Independent	**Knowledge of organic agriculture principles**	People’s awareness level on the principles of organic agriculture include health, ecology, fairness and care.
Independent	**Moral norms**	They are considered as a pattern for public expectation of behavior rising from group values. In the present study, moral norms are the society’s expectations, as well as farmers’ self-expectation of performing environmental activities as a moral commitment in a particular situation (for instance, not using chemical inputs).
Independent	**Responsibility of pro-environmental behavior**	Responsibility includes honesty, respect, responsiveness, justice, and courage. In addition, the responsibility of behavior is commitment to perform personal acts and accept the consequence of those acts. In the study, the responsibility of behavior means accepting the consequences of agricultural activities by farmers and trying to decrease undesired environmental consequences.
Independent	**Environmental identity**	It is the internal and mental relationship with natural and non-human environment based on historical record, emotional affiliations, and similarities which affect human behavior with the world. Overall, human belongs to environment and that nature is part of him.
Independent	**Attitude to negative consequences of the conventional agriculture**	Evaluating the conventional agriculture consequences negatively or positively, as well as the attitude toward the outcomes of this agriculture system
Independent	**Social norms**	The norms are considered as the regulations conducting behaviors. In the present study, they refer to farmers’ perception of social expectations and pressures to show pro-environmental behaviors.
Intermediate	**Perceived control of pro- environmental behavior**	People’s perception of their control on personal actions to protect the environment by choosing and being willing to do pro-environmental activities such as organic agriculture
Intermediate	**Attitude to the environment**	People’s belief in environmental problems and concerns about these problems or a complicated and multi-dimensional concept including negative and positive emotions on the environment, and a mental state which affects people’s selections on the environment
Intermediate	**Intention for organic agriculture**	The intention means providing the personal instructions to perform a set of particular actions for achieving planned aims. Therefore, it is defined as individual’s intention to plan for eliminating each type of harmful and chemical inputs in agricultural activities, as well as adopting the organic agriculture.
Dependent	**Adoption of organic agricultural activities**	Implementing the principles and different techniques of organic agriculture properly and practically such as replacing compost, vermicompost and green fertilizers instead of chemical inputs, no-tillage technology, etc.

(2018; Rehfus 2003; Sheeran and Webb 2016; Clayton 2003; Malek-Saeidi et al. 2012; Tatlıdil et al. 2009; King and Ilbery 2012; Rigby and Cáceres 2001; Stryker and Burke 2000)

## Results and discussion

### The mean of variables

The mean and standard deviation of research variables have been shown in Table 3. The mean of “intention for organic agriculture” is 12.71, which means that there is a relatively high intention to organic agriculture among the farmers. The mean of “knowledge of organic agriculture principles” is 43.22, which means that farmers’ knowledge on the principles of organic agriculture is moderate to high due to the score range of this variable. In addition, the mean of “attitude to negative consequences of the conventional agriculture” is 30.14. In fact, the farmers have high attitude to negative consequences of the conventional agriculture. Further, the farmers are in a desirable situation based on environmental identity with the mean of 33.46. In other words, the farmers are in a moderate situation regarding responsibility of pro-environmental behavior (19.9) and moral norms (26.6) to organic activities.

**Table 3:** The description of model variables

Variable	Scale	Mean	SD
Environmental identity	5-10	33.46	4.62
Intention for organic agriculture	3-15	12.71	1.82
Knowledge of organic agriculture principles	13-65	43.22	5.89
Adoption of organic agricultural activities	15-75	36.31	7.91
Attitude toward the environment	5-25	19.72	2.77
Perceived control of pro-environmental behavior	7-35	13.49	5.65
Social norms	10-50	30.68	7.1
Responsibility of environmental behavior	6-30	19.92	3.37
Moral norms	9-45	36.64	5.11
Attitude to negative consequences of the conventional agriculture	9-45	30.14	5.9

### Adoption of the organic agricultural activities based on the degree of education

**Fig. 3:**
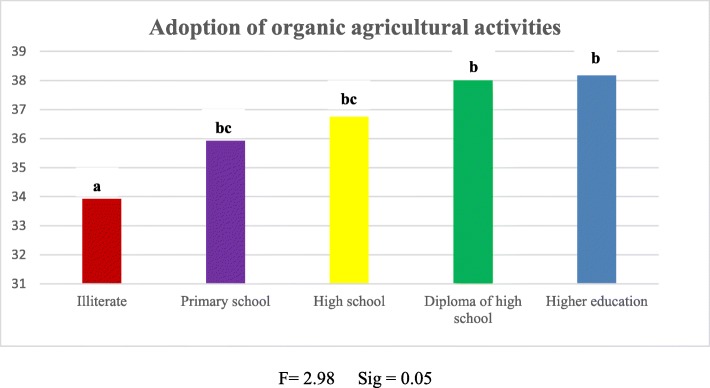
Mean comparison of adoption of organic agricultural activities based on the degree of education

The results of ANOVA demonstrated a significant difference between degree of education and adoption of the organic agricultural activities by farmers (F = 2.98, *P* = 0.05). Considering the LSD test, a significant difference was observed between illiterate farmers and those with elementary, secondary and graduated education (Fig. 3). In other words, the farmers with high education degree (secondary and higher) perform more organic cultural activities. Therefore, the degree of education is considered as an influential factor for performing organic agricultural activities.

### Adoption of organic agricultural activities based on the perceived control of pro-environmental behavior

Based on the results of ANOVA in Table 4, a significant difference was reported between various levels of perceived control of pro-environmental behavior for performing organic agricultural activities, and the adoption of them (F = 7.13, *P* = 0.04). Regarding the results of LSD test, the farmers who control the needed resources and facilities for organic agricultural activities perform these activities more in their lands.

**Table 4:** The results of ANOVA for organic agricultural activities given the perceived control of the pro-environmental activities

	Mean	SD	F	Sig
Low	34.6^*b*^	8.39	7.13	0.04
Moderate	35.72^*ab*^	7.09		
High	38.16^*a*^	8.56		

The score range is 15–75

### Adoption of the organic agricultural activities given the areas under cultivation

The results of ANOVA demonstrated a significant difference between the areas under cultivation and adoption of the organic agricultural activities (F = 8.19, *P* = 0.001). Regarding LSD test, there is a significant difference between the farmers with low areas under cultivation and those with high and moderate areas under cultivation, although no difference was observed between the farmers with moderate areas under cultivation and those with high areas under cultivation (Table 5). Therefore, the results showed that increasing the areas under cultivation leads to a decrease in the adoption of organic agricultural activities. The results can be justified by considering low pro-environmental behavior control and farmers’ low access to production factors for adoption of the organic agricultural activities.

**Table 5:** The results of ANOVA for organic agricultural activities by the areas under cultivation

	Mean	SD	F	Sig.
High	33.79^*b*^	8.73	8.19	0.001
Moderate	35.59^*ab*^	7.36		
Low	40.63^*a*^	6.95		

The score range is 15–75

### Correlation between variables and accepting the organic agricultural activities

Based on Table 6, the attitude to negative consequences of the conventional agriculture has a positive and significant relationship with adoption of the organic agricultural activities (r = 0.259, *P* = 0.001). Increasing the awareness of negative consequences of the conventional agriculture increases the organic agricultural behavior among farmers. According to findings, the attitude to the environment is positively related to adoption of the organic agricultural activities (r = 0.238, P = 0.001). Enriching the farmers’ attitude to the environment enhances adoption of the organic agricultural activities. In addition, a positive and significant relationship was reported between the knowledge of organic agriculture principles and organic agricultural activities (r = 0.346, P = 0.001). Enhancing the knowledge of organic agriculture principles improves adoption of the organic agricultural activities. Rational selection theory emphasizes the relation between attitude and knowledge and behavior outbreak (Ajzen 2001). Further, a significant and positive relationship was observed between perceived control of the pro-environmental behavior and adoption of the organic agricultural activities (r = 0.200, P = 0.001). In other words, everyone who has more access to production factors for organic agricultural activities such as loan and other facilities demonstrates more organic agricultural behaviors. The theory of planned behavior has been focused on the correlation of perceived control of pro-environmental behavior and adoption activities (Lane and Potter 2007).

**Table 6:** The correlation matrix of research variables and organic farming adoption

	Variables	1	2	3	4	5	6	7	8	9	10
1	Intention to organic agriculture	1									
2	Moral norm	0.112	1								
3	Responsibility of pro-environmental behavior	0.212^**^	0.219^**^	1							
4	Attitude toward negative consequences of conventional agriculture	0.348^**^	0.392^**^	−0.037	1						
5	Knowledge of organic farming principles	0.018	0.095	0.175^*^	0.036	1					
6	Social norm	0.115	0.182^*^	0.018	0.007	0.191^*^	1				
7	Attitude toward environment	0.041	0.221^**^	0.176^*^	− 0.06	0.134	0.255^**^	1			
8	Perceived control of pro-environmental behavior	0.064	0.191^*^	0.308^**^	0.253^**^	−0.104	0.074	−0.243^*^	1		
9	Environmental identity	0.186^*^	0.362^**^	0.112	0.331^**^	0.177^*^	0.211^**^	0.86	0.201^**^	1	
10	Adoption of organic farming	0.132^*^	0.370^**^	0.305^**^	0.259^*^	0.346^**^	0.179^*^	0.238^**^	0.200^**^	0.505^**^	1

*Significant level at 0.05

*Significant level at 0.01

Furthermore, a positive and significant correlation was achieved between social norms and adoption of organic agriculture (r = 0.179, *P* = 0.05). In fact, organic agriculture behavior improves by enhancing the social norms. In addition, moral norms have a significant relationship with adoption of the organic agricultural activities (r = 0.370, *P* = 0.01). There is significant correlation between improvement of ethical and social norms among farmers and environmental behaviors by organic activities due to the “Norm Activation Model” and “Value-Belief-Norm” theories (Stern 2000). In addition, there is a positive and significant correlation between environmental identity and adoption of organic agricultural activities (r = 0.505, P = 0.05). Thus, organic agricultural activities are increased by enhancing the farmers’ environmental identity. Most environmental sociology studies have been focused on the relations between environmental identity and environmental behaviors such as organic activities in farming (Burke 1991; Rezaei-Moghaddam and Fatemi 2013). Intention to organic agriculture has a positive and significant correlation with adoption of the organic agricultural activities (r = 0.132, P = 0.05). Therefore, enhancing intention to organic agriculture increases organic agricultural activities.

### Analyzing the effects of the variables on adoption of the organic agricultural activities

Estimation of measuring models, including test results of the model is listed in Table 7. As it could be seen, the goodness-of-fit measures indicated an adequate fit.

**Table 7:** Goodness of fit measures

Model fit indices	Recommended values	Proposed model
Df	–	5
X^2^	–	6.33
X^2^ /df	5≥	1.26
GFI	0.90≤	0.98
AGFI	0.80≤	0.91
CFI	0.90≤	0.98
NFI	0.90≤	0.99
RMSEA	0.1≥	0.04

The total and direct effects of all variables on perceived control of pro-environmental behavior as intermediate variable have been shown in Table 8. The variables which directly and negatively affect the perceived control of the pro-environmental behavior include the moral norm with direct effect of − 0.15 and social norm with direct effect of − 0.17. The findings suggest that social expectations to perform organic agricultural activities do not aim to encourage using the resources for adoption of these activities. Therefore, internal and moral commitment does not aim to use these resources.

**Table 8:** The direct and total standardized effects of variables on perceived control of pro-environmental behavior

Variables	Direct effect	Total effect
Moral norm	−0.15	−0.15
Social norm	−0.17	−0.17
Knowledge about organic farming principles	0.08	0.08
Attitude toward negative consequences of conventional agriculture	0.11	0.11
Environmental identity	−0.11	−0.11
Environmental behavior responsibility	0.07	0.07

The direct and total effects of research variables on another intermediate variable, attitude toward environment, are indicated in Table 9. Social norm, responsibility of pro-environmental behavior, and environmental identity variables influence farmers’ attitude to the environment with the direct effect of 0.38, 0.24 and 0.21, respectively. The other effective factors on the attitude to the environment are the attitude toward negative consequences of conventional agriculture, knowledge of organic agriculture principles, and social norms with direct effects of 0.18, 0.16 and 0.19, respectively. Among the effective variables, moral norms have the greatest effect on attitude to the environment. Responsibility of pro-environmental behavior and environmental identity have a significant positive and direct effect on farmers’ environmental attitude. There is considerable focus on the important role of norms, perceived control of pro-environmental behavior and environmental identity of people in the theories of Norm Activation Model, Value-Belief-Norm as well as environmental sociology (Klockner 2013; Rezaei-Moghaddam and Fatemi 2013).

**Table 9:** The direct and total standardized effects of variables on attitude toward environment

Variables	Direct effect	Total effect
Moral norm	0.38	0.38
Social norm	0.19	0.19
Knowledge about organic farming principles	0.16	0.16
Attitude toward negative consequences of conventional agriculture	0.18	0.18
Environmental identity	0.21	0.21
Responsibility of pro-environmental behavior	0.24	0.24

**Fig. 4:**
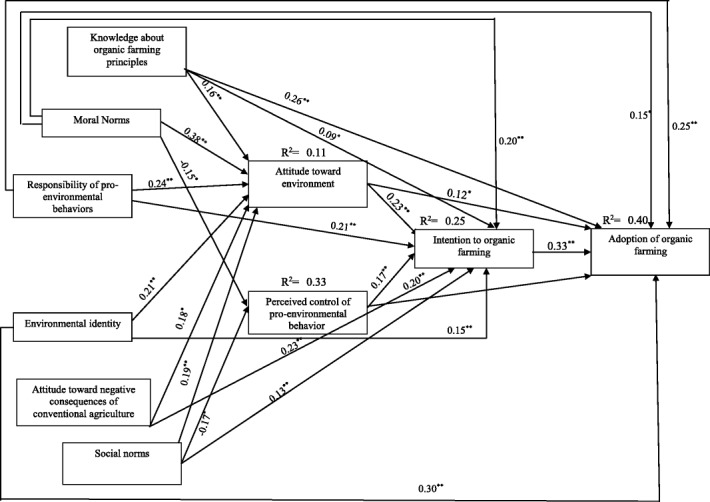
Influencing factors of organic farming adoption due to path analysis results

The direct, indirect and total effects of research variables on intention to organic farming adoption have been shown in Table 10. According to Fig. 4, attitude to negative consequences of the conventional agriculture, attitude to the environment and moral norm with direct effect of 0.23, 0.23 and 0.20, respectively, as well as the responsibility of pro-environmental behavior with significant and direct effect of 0.21 are regarded as some effective factors in intention for adoption of organic agriculture. In addition, perceived control of pro-environmental behavior with direct effect of 0.17, environmental identity with direct effect of 0.15, social norm with direct effect of 0.13 and knowledge of organic agriculture with direct effect of 0.09 influence the intention for organic agriculture. According to rational selection and planned behavior theories, there is great emphasis on the role of attitudes and perceived control of pro-environmental behavior on intentions and activities of individuals (Ajzen 2001). It is also mentioned on moral and social norms, environmental identity and behavior responsibility due to the different environmental sociological models (Rezaei-Moghaddam and Fatemi 2013; Stryker and Burke 2000).

**Table 10:** The direct and total standardized effects of variables on intention to organic farming adoption

Variables	Direct effect	Indirect effect	Total effect
Moral norm	0.2	−0.07	0.13
Social norm	0.13	0.02	0.15
Knowledge about organic farming principles	0.09	0.05	0.14
Attitude toward negative consequences of conventional agriculture	0.23	0.04	0.27
Environmental identity	0.15	0.03	0.18
Responsibility of pro-environmental behavior	0.21	0.05	0.26
Perceived control of pro-environmental behavior	0.17	–	0.17
Attitude toward environment	0.23	–	0.23

Table 11 displays the results of effects of intermediate and independent variables on adoption of the organic agricultural activities by the farmers. The most influential effects of intermediate variable are related to the intention for adoption of organic agriculture with direct effect of 0.33 and then, knowledge of organic agriculture principles and perceived control of pro-environmental behavior with the direct effect of 0.26 and 0.20, respectively. The knowledge and perceived control of pro-environmental behavior are the two main factors of individual’s behavior due to the rational selection and planned behavior theories (Ajzen and Fishbein 1980). Attitude to the environment and moral norm variables directly effect on adoption of the organic agriculture with direct effect of 0.12 and 0.15, respectively. Responsibility of pro-environmental behavior has a significant direct effect (0.25) on adoption of the organic agricultural activities. In addition, environmental identity has a significant and direct effect on adoption of the organic agricultural activities (0.30). The effect of two variables of norms and behavior responsibility have been emphasized in different theories especially in environmental sociology (Rigby and Cáceres 2001; Rezaei-Moghaddam and Fatemi 2013). Knowledge of organic agriculture principles, moral norm, responsibility of pro-environmental behavior, environmental identity, attitude to negative consequences of the conventional agriculture, social norm, attitude to the environment and perceived control of pro-environmental behavior have indirect effects on adoption of the organic agricultural activities, as shown in Table 11. Further, the attitude to negative consequences of the conventional agriculture (0.12) has an indirect effect on adoption of the organic agricultural activities by the intermediate variables. Furthermore, the attitude to protect the environment and knowledge of organic agriculture principles (0.08) have an indirect effect on adoption of the organic agricultural activities.

**Table 11:** The total, direct, and indirect standardized effects of the variables on adoption of the organic agricultural activities

Variable	Direct effect	Indirect effect	Total effect
Moral norm	0.15	0.03	0.18
Social norm	0.06	0.04	0.10
Knowledge about organic farming	0.26	0.08	0.34
Attitude toward negative consequences of conventional agriculture	0.08	0.12	0.20
Environmental identity	0.30	0.04	0.034
Responsibility of pro-environmental behavior	0.25	0.05	0.30
Perceived control of pro-environmental behavior	0.20	0.05	0.25
Attitude toward environment	0.12	0.08	0.20
Intention to adoption of organic agriculture	0.33	0.00	0.33

## Conclusion

The conventional production structure which is based on high chemical inputs such as chemical fertilizers and pesticides in producing agricultural products has been criticized. Observing the undesired effects of the conventional agriculture in the world results in focusing on the immediate need for developing agricultural techniques which are environmentally and socio-economically sustainable. Organic agriculture is considered as one of the most critical alternative agricultural systems to produce healthy food without any chemicals. The studied farmers are highly aware of the negative consequences of the conventional agriculture. There is a strong intention to perform organic agriculture among the farmers. The farmers who have suitable access to resources and needed facilities to perform organic activities do more of these activities in their land. Paying attention by policy-makers in Iran to some mental and personal features such as environmental identity and trying to increase people’s attachment to the surrounding nature by communication and extension channels could be a fundamental approach for improving the farmers’ intention to adopt new protective methods of the environment such as organic.

The farmers’ intention to adopt organic agriculture, environmental identity, responsibility of pro-environmental behavior, and moral norms can influence adoption of the organic agricultural activities critically. Also, farmers’ knowledge of organic agriculture principles, perceived control of pro-environmental behavior, and their access to resources and facilities are vital to perform organic activities. The environmental sociology specialists emphasize the norm effect, especially the farmers’ internal commitment, as well as the behavior responsibility. In fact, the agriculture extension policy-makers in Iran should invest in changing the farmers’ mental, behavioral, and social features, which play an essential role in protecting the environment and encouraging the farmers to adopt and use nature friendly techniques by implementing organic methods. The various studies, especially in the environmental sociology, emphasize the role of farmers’ knowledge and access to resources for the outbreak of pro-environmental behaviors. Therefore, more attention should be paid by Iranian policy-makers to providing needed backgrounds such as increasing farmers’ knowledge and awareness of organic agriculture principles and methods (such as new techniques of ASD in preparing farmland for decreasing pests and diseases, as well as combined and biological methods against pests and disease), and after changing the farmers’ environmental attachment fundamentally, the responsibility of behavior consequences, and behavioral control. To this aim, the audience access to required resources and facilities for implementing the organic techniques should be regarded and facilitated.

Prediction and improvement of behaviors related to environment should be and appear to be evolving as a universal priority. Analysis of different theories and models indicates that attitudes and beliefs may not lead in a straightforward way to environmental behaviors. Conventional models fail to incorporate the dynamics of interactions between and impact of individual and communal human behavior and the environment. Social factors influence individual attitude and intention, thereby impacting behavior toward environment. A more robust and systematic sociological study of environmental issues seems to be universally meritorious and very timely.

## Data Availability

The datasets generated and/or analyzed during the current study are not publicly available due [Because all of the data was gathered by the research team] but are available from the corresponding author on reasonable request.

## Abbreviations

PBC: Perceived Behavioral Control
TPB: Theory of Planned Behavior
VBN: Value-Belief-Norm
REB: Responsible Environmental Behavior
NEP: New Ecology Paradigm
ANOVA: Analysis of Variance
LSD: Least Significance Difference
Sig.: Significance Level
SD: Standard Deviation
df: Degree of freedom
NFI: Normed Fit Index
CFI: Comparative Fit Index
GFI: Goodness-of-Fit Index
AGFI: Adjusted Goodness of Fit Index
RMSEA: Root Mean Square Error of Approximation
